# Primary splenic histiocytic sarcoma complicated with prolonged idiopathic thrombocytopenia and secondary bone marrow involvement: a unique surgical case presenting with splenomegaly but non-nodular lesions

**DOI:** 10.1186/1746-1596-7-143

**Published:** 2012-10-17

**Authors:** Sohsuke Yamada, Takashi Tasaki1, Naoko Satoh, Atsunori Nabeshima, Shohei Kitada, Hirotsugu Noguchi, Kozue Yamada, Morishige Takeshita, Yasuyuki Sasaguri

**Affiliations:** 1 Department of Pathology and Cell Biology, School of Medicine, University of Occupational and Environmental Health, Kitakyushu, 807-8555, Japan; 2 Department of Pathology, Kyushu Kosei-Nenkin Hospital, Kitakyushu, 806-8501, Japan; 3 Department of Urology, School of Medicine, University of Occupational and Environmental Health, Fukuoka, 814-0180, Japan; 4 Department of Pathology, Faculty of Medicine, Fukuoka University, Fukuoka, 814-0180, Japan

**Keywords:** Thrombocytopenia, Spleen, Splenomegaly, Histocytic sarcoma (HS), Hemophagocytosis

## Abstract

**Abstract:**

A 67-year-old Japanese female was followed up due to prolonged idiopathic thrombocytopenia with non-response to steroid therapy for 4 years, but recent progressive pancytopenia, hypo-albuminemia, and hypo-γ-globulinemia were presented. An abdominal CT scan revealed heterogeneously enhanced splenomegaly without any nodular lesions. A splenectomy was performed, and gross examination showed markedly hyperemic red pulp, weighing 760 g, accompanied by multiple foci of peripheral anemic infarction. Surprisingly, microscopic findings exhibited a diffuse proliferation of medium-sized to large tumor cells having pleomorphic nuclei, prominent nucleoli, and abundant eosinophilic cytoplasm, predominantly within the sinuses and cords of the red pulp, which occasionally displayed conspicuous hemophagocytosis and vascular permeation. In immunohistochemistry, these atypical cells were specifically positive for CD68 (KP-1), CD163, and lysozyme, which was consistent with histiocytic sarcoma (HS) of the spleen. Subsequently, section from the aspiration of bone marrow showed infiltration of the neoplastic cells associated with erythrophagocytosis 2 months after the operation, but never before it. Therefore, primary splenic HS presenting with secondary bone marrow involvement was conclusively diagnosed. Since early diagnosis and treatment are necessary for the HS patients with poor outcomes, splenic HS should be considered as a differential diagnosis in cases with chronic thrombocytopenia and splenomegaly.

**Virtual slides:**

The virtual slide(s) for this article can be found here: http://www.diagnosticpathology.diagnomx.eu/vs/1009474924812827

## Background

By definition, histiocytic sarcoma (HS), a rare hematopoietic neoplasm, but a known entity, representing as less than 0.5% of all non-Hodgkin’s lymphoma, is malignant proliferation of cells presenting morphologic and immunophenotypic characteristics of mature tissue histiocytes [1,2]. HS is known to often present as localized disease confined to the intestinal tract, skin, lymph node, or soft tissues [1-3], however, primary HS of the spleen has been an exceedingly rare. Mathé G et al. first proposed the term ‘HS’ tentatively in 1970 [4]. In contrast, since its description was strictly based on the histologic similarities of the tumor cells to macrophages, this diagnostic challenge is further complicated as it is critical to clearly distinguish HS from other histiocytic processes, from benign to malignant, such as hemophagocytic syndrome, malignant histiocytosis, and acute monocytic leukemia [5-7]. Despite that, the detailed evaluation of HS depends especially on the verification of histiocytic lineage and the exclusion of other malignancies, including anaplastic large cell lymphoma, diffuse large B-cell lymphoma, poorly differentiated carcinoma, malignant melanoma, follicular dendritic cell sarcoma, or interdigitating dendritic cell sarcoma, by way of thorough immunohistochemical examination [1-3,8,9]. In this context, it is very difficult to interpret HS papers published more than 25 years ago, i.e., before the development and widespread use of immunohistochemical techniques and the availability of molecular genetic tools [3]. Actually, until now, the number of ‘true’ cases reported as primary splenic HS in the English literatures is only few, up to 7, and most recent references are from 2008 within our investigation [10-14], summarized in Table 1, and its clinicopathological features have not been well described. Based on this Table 1, HS of the spleen can be potentially lethal condition that might remain asymptomatic or closely related with chronic thrombocytopenia for a long time [11-13]. Even while diagnostic imaging findings have not been also characterized satisfactorily, primary splenic HS cases often show multi-nodular or nodular lesions in the enlarged tissues of spleen [10-14]. The patients of splenic HS have a poor prognosis due to aggressive behavior with liver or bone marrow infiltration, even though a splenectomy might induce temporary remission [11,14]. Therefore, early accurate diagnosis and treatment before disseminated proliferation of the HS tumor cells should improve their survival rates.

**Table 1:** Comparison of the main clinical features among the reported cases with primary splenic HS, including ours

**Case [reference]**	**Age**	**Sex**	**CC**	**Imaging or Gross features**	**Weight of spleen**	**Therapy**	**Prognosis**	**Involvement**
1 [10]	38	m	Weakness	multinodular	264 g	S	-	-
2 [11]	29	m	edema	nodular	735 g	S(R+C)	5Y1M	Liver
3 [11]	60	m	thrombocytopenia	nodular	610 g	S+C	1Y6M	Liver, BM
4 [11]	66	f	edema, anemia	multinodlar	750 g	R+S+C	2Y6M	Liver, BM
5 [12]	71	f	thrombocytopenia	multinodlar	470 g	C	6 M	Hilum LN
6 [13]	82	f	Evans syn,	multinodular	110 g	R	1 M	Liver
7 [14]	58	f	anemia	nodular	800 g	R+S+C	3 M	-
Present case	67		thrombocytopenia	non-nodula	760 g	S	6 M	BM

*CC* chief complaint, *S* surgery (splenectomy), *R* radiation, *C* chemotherapy, *Y* year, M month, *BM* bone marrow, *LN* lymph node.

Indeed, this is not the first case reported. Despite that, we reported a very rare and unique surgical case of primary splenic HS complicated with prolonged idiopathic thrombocytopenia, progressive pancytopenia, hypo-albuminemia, hypo-γ-globulinemia, and secondary bone marrow involvement, which grossly presented as splenomegaly with multiple peripheral infarction but without any nodular foci.

## Materials and methods

The patient was a 67-year-old Japanese female. The specimen after fixation in 10% neutral buffered formalin was embedded in paraffin for histological or immunohistochemical examinations. All immunohistochemical stainings were carried out using Dako Envision kit (Dako Cytomation Co., Glostrup, Denmark) according to the manufacturer’s instructions, and using commercially available prediluted monoclonal antibodies against the following antigens: CD1a (Immuno Tech. Co., Ltd., Osaka, Japan, diluted 1:2), CD3 (Dako, diluted 1:1), CD4 (Dako, diluted 1:1), CD5 (NOVOCASTRA laboratories Ltd., Newcastle, United Kingdom, diluted 1:25), CD8 (Nichirei Biosciences Inc., Tokyo, Japan, diluted 1:1), CD10 (NOVOCASTRA, diluted 1:20), CD20 (Dako, diluted 1:200), CD21 (Dako, diluted 1:10), CD23 (Nichirei, diluted 1:1), CD30 (Dako, diluted 1:40), CD45 (Dako, diluted 1:400), CD45RO (UCHL-1; Dako, diluted 1:200), CD68 (KP-1; Dako, diluted 1:100), CD79a (Dako, diluted 1:50), CD163 (Leica Microsystems, Wetzlar, Germany, diluted 1:200), lysozyme (Dako, diluted 1:600), S-100 protein (Dako, diluted 1:900), fascin (Dako, diluted 1:50), bcl-2 (Dako, diluted 1:30), myeloperoxidase (MPO; Dako, dilutede 1:500), EMA (Dako, diluted 1:100), Cam5.2 (Becton Dickinson Immunocytometry Systems, San Jose, CA, diluted 1:1), and HMB45 (Enzo Life Sciences Ltd., New York, diluted 1:100). Epstein-Barr virus (EBV) infection status was analyzed by in situ hybridization for EBV-encoded RNAs using an Epstein-Barr Early RNA Probe Reagent (EBER; Roche Applied Science, Lewes, UK). However, no chromosome studies have been performed.

### Case presentation

**Figure 1:**
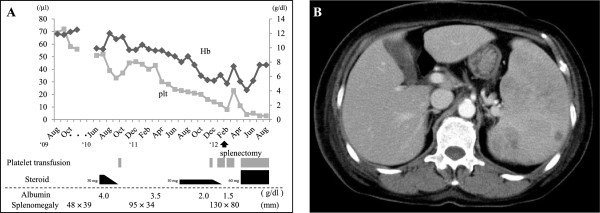
**Summary of various clinical data before and after splenectomy, and the finding of an abdominal CT scan at surgery.** (**A**) This patient showed prolonged idiopathic thrombocytopenia presenting with increased splenomegaly and non-response to steroid therapy. In addition, progressive pancytopenia, hypo-albuminemia, and hypo-γ-globulinemia coexisted. (**B**) The abdominal CT scanning revealed heterogeneously enhanced, severe splenomegaly without evidence of mass or nodular lesions.

This patient had 4-year-history of idiopathic thrombocytopenia with non-response to steroid therapy and progressive pancytopenia half year before a splenectomy (Figure 1A). There was no history of immunosuppressive disorders, use of immunosuppressive medications, or unusual infections. The abdominal CT scanning showed heterogeneously enhanced and growing splenomegaly without evidence of mass or nodular lesions, measured approximately 130 × 80 mm in diameter (Figure 1B). Retrospectively, the clinical imaging examination also demonstrated mild splenomegaly, measured 48 × 39 mm or 95 × 34 mm in diameter 3 or 2 years before surgery, respectively (Figure 1A). In laboratory data on admission, blood cell counts revealed pancytopenia; white blood cell count (WBC) was 1,300/mm^3^; hemoglobin (Hb) was 5.7 g/dL; and platelet count (Plt) was 8,000/mm^3^ (Figure 1A). Biochemistry showed almost within normal limits, except for low levels of total protein (TP; 4.9 g/dL), albumin (Alb; 1.6 g/dL), and γ-globulin (γ-gl 0.6 g/dL) (Figure 1A), whereas high level of C-reactive protein (CRP; 4.07 mg/dL). Serum interleukin 2 receptor (sIL-2R) level as a tumor marker was only increased up (1306.0 U/mL). Physical examination on admission demonstrated no remarkable change, except for mild weakness. Since the clinician highly suspected hypersplenism, a splenectomy was performed. The patient was dead due to secondary bone marrow involvement of the HS tumor cells, as described more in detail below. Autopsy could not be examined because of the family’s objection. The clinical findings including laboratory examination and therapy for 4 years are summarized in Figure 1A until his death 6 months after the surgery.

### Pathological findings

Before the operation, the clinician and we pathologists had performed and examined bone marrow aspiration 5 times for 2 and half years (September in 2009, September and December in 2010, December in 2011, and January in 2012), histologically showing the tissues of normo- to hyper-cellular marrow (cellularity: 50-70%) composed of 3 series of hematopoietic cells with a mildly increasing number of bland-looking megakaryocytes (4-8/1HPF). There was no evidence of monotonous proliferation of atypical or blastoid cells in the bone marrow specimens (not shown).

**Figure 2:**
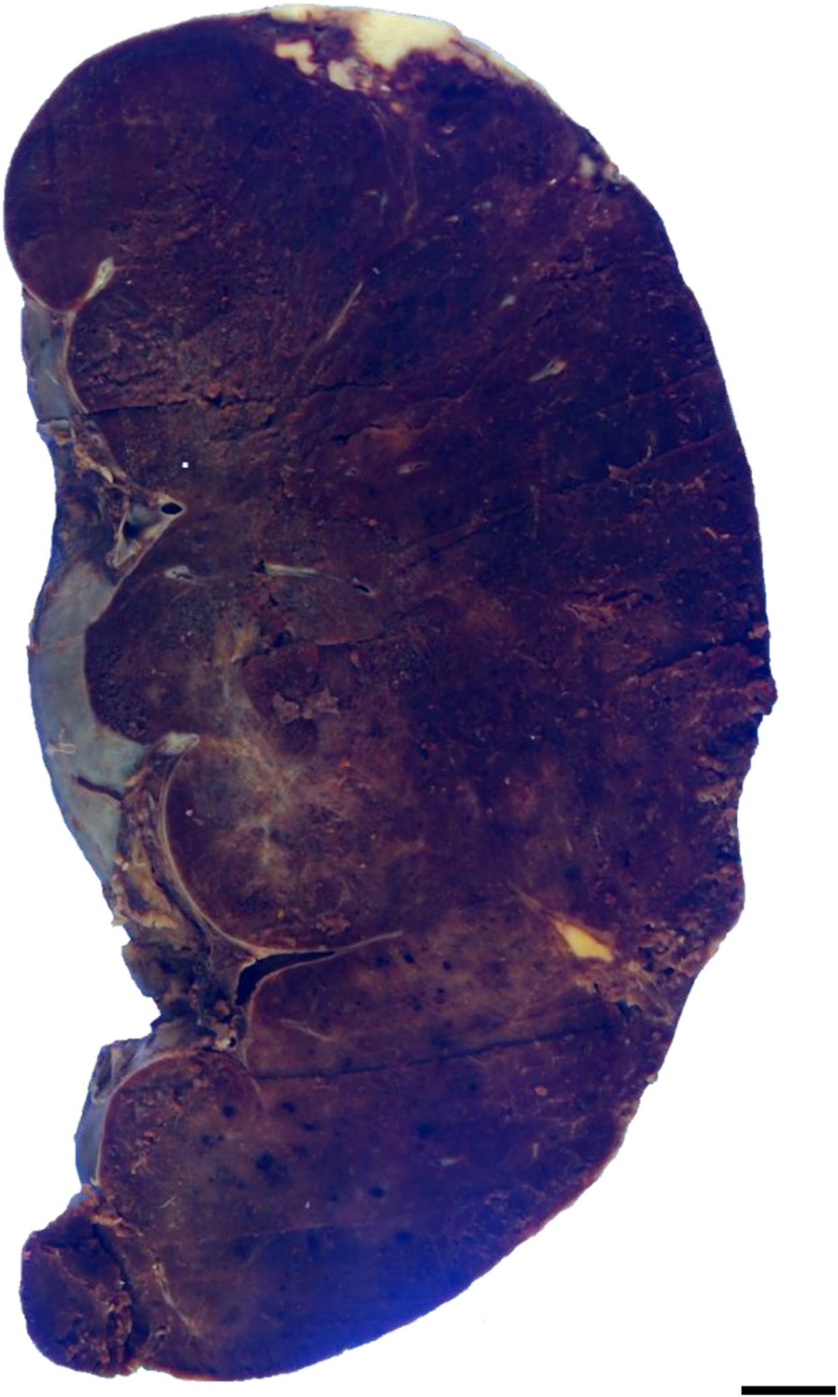
**Gross examination of splenic HS from the resected specimen.** On gross examination of the splenomegaly, the cut surface showed multiple foci of peripheral anemic infarction, but not any mass or nodular lesions, which predominantly looked dark-red in colour associated with congestive red pulp and depleted white pulp. Bar = 10 mm.

On gross examination of the surgical specimen, the spleen was markedly enlarged, measuring 145 × 120 × 80 mm and weighing 760 g. The cut surface revealed multiple foci of peripheral infarction but not mass or nodular lesions, which predominantly looked dark-red in colour associated with congestive red pulp and depleted white pulp (Figure 2). A scanning magnification of it showed markedly hyperemic red pulp, coexisted with peripheral anemic infarcts and hemorrhage, but not encapsulated tumor-like lesions (not shown).

**Figure 3:**
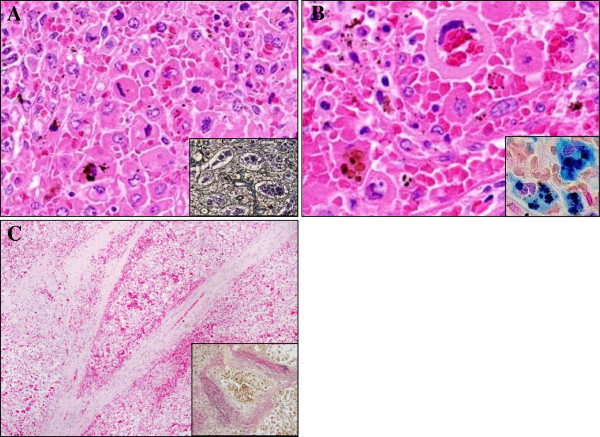
**Histological examination of splenic HS.** (**A**) The spleen predominantly showed a diffuse proliferation of medium-sized to large tumor cells having mildly hyperchromatic and pleomorphic nuclei, prominent nucleoli, and abundant eosinophilic or clear cytoplasm, mainly within the sinuses and cords of the red pulp, highlighted by silver impregnation (inset) (H&E stains, Original magnification × 200). (**B**) On high-power view, these large tumor cells exhibited apparent hemophagocytosis of red blood cells or mononuclear lymphocytes (H&E stains, Original magnification × 400). Hemosiderin pigments within the large cells confirmed erythrophagocytosis by Berlin-blue staining (inset). (**C**) Vascular invasion of these tumor cells was noted in the trabecular artery, very likely inducing multiple foci of peripheral anemic infarction (H&E stains, Original magnification × 40). This could make a clear contrast by elastica van Gieson (EVG) staining (inset).

**Figure 4:**
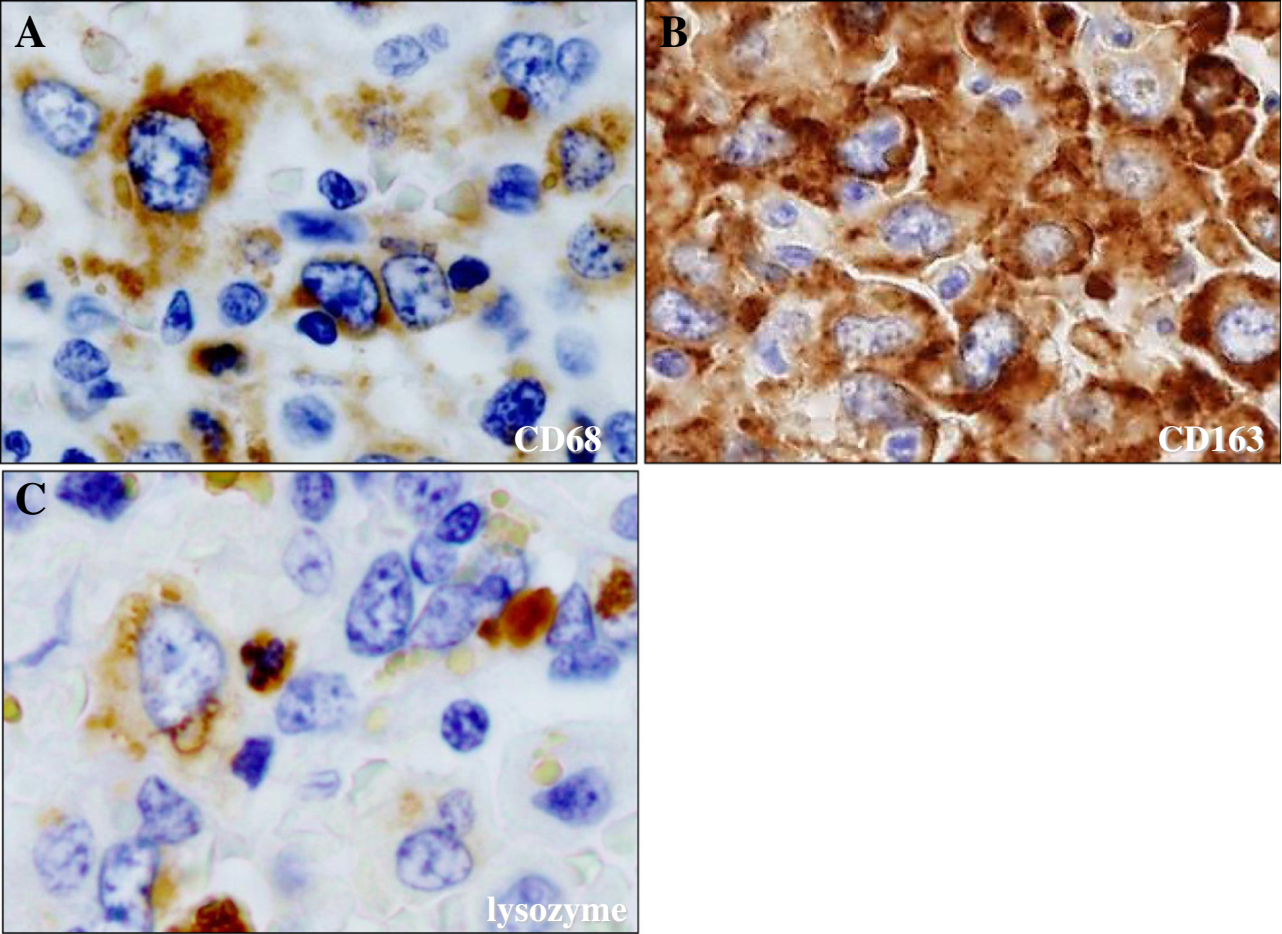
**Immunohistochemical examination for the tumor cells of splenic HS. (A)(B)(C)** These large tumor cells were specifically positive for CD68 (KP-1) (**A**), CD163 (**B**), and lysozyme (**C**), and many reactive histiocytes were also positive for CD163 (Original magnification × 400).

Microscopically, the spleen predominantly displayed a diffuse proliferation of medium-sized to large tumor cells having mildly hyperchromatic and pleomorphic nuclei, prominent nucleoli, and abundant eosinophilic or clear cytoplasm, mainly within the sinuses and cords of the red pulp, highlighted by silver impregnation (Figure 3A). However, neither nodular lesions nor fibrous encapsulation were evident with Masson’s trichrome staining (data not shown). On high-power view, these large cells occasionally showed apparent hemophagocytosis of red blood cells or mononuclear lymphocytes (Figure 3B). Hemosiderin pigments in the hemophagocytic tumor cells confirmed erythrophagocytosis with Berlin-blue staining (Figure 3B). On the other hand, vascular invasion of the tumor cells was sometimes noted in the trabecular artery, making a clear contrast by elastica van Gieson (EVG) staining (Figure 3C). It was suggested that vascular permeation could induce multiple foci of peripheral anemic infarction associated with hemorrhage. Additionally, adjacent to these infarcts, a very small amount of the existing splenic tissue was remarkably compressed. In immunohistochemistry, these large cells were specifically positive for CD68 (KP-1) (Figure 4A), CD163 (Figure 4B), lysozyme (Figure 4C), S-100 protein, and CD45, and many reactive histiocytes were also positive for CD163 and S-100 protein. Whereas, the tumor cells were negative for CD1a, CD3, CD4, CD5, CD8, CD10, CD20, CD21, CD23, CD30, CD45RO (UCHL-1), CD79a, fascin, bcl-2, myeloperoxidase (MPO), EMA, Cam5.2, and HMB45. All immunohistochemical profile of the tumor cells is summarized in Table 2. By contrast, CD8 staining confirmed that proliferating tumor cells were existed within the sinuses and cords of the red pulp, accompanied focally by the destruction of the sinuses (data not shown). On the other hand, in situ hybridization for EBV-encoded RNAs was negative. Based on all these features, we made a diagnosis of histiocytic sarcoma (HS) of the spleen.

**Table 2:** Immunohistochemical profile of the tumor cells in our splenic HS case

**positive**	**negative**	
CD68 (KP-1)	CD1a	CD30
CD163	CD3	CD45RO
lysozyme	CD4	CD79a
S-100 protein	CD5	fascin
CD45 (LCA)	CD8	bcl-2
	CD10	MPO
	CD20	EMA
	CD21	Cam 5.2
	CD23	HMB45

**Figure 5:**
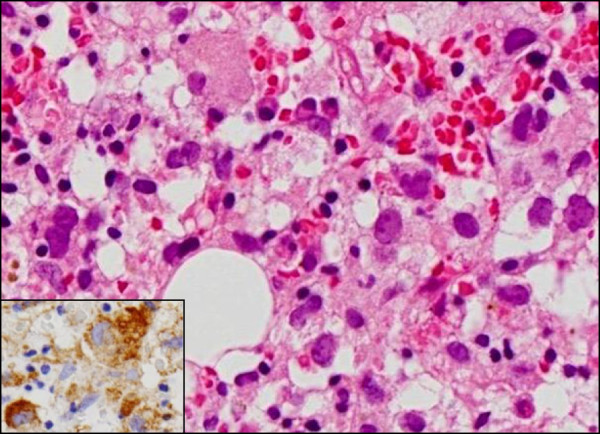
**Histological and immunohistochemical examination of secondary bone marrow involvement of primary splenic HS.** 2 months after the splenectomy, the tumor cells infiltrated the bone marrow tissue (H&E stains, Original magnification × 200). Immunohistochemical CD68 staining (inset) could make it much easier to understand the microscopic findings of HS involvement in the bone marrow.

Furthermore, 2 months after the splenectomy, the HS cells partially infiltrated the bone marrow (Figure 5A), coexisting with a substantial amount of 3 series of normocellular marrow including a significantly increasing number of megakaryocytes. Immunohistochemical CD68 (Figure 5B) and S-100 protein stainings could make it much easier to understand the microscopic findings of HS involvement in the bone marrow. The above clinical and pathological findings indicated that this case was really primary splenic HS presenting with secondary bone marrow involvement.

## Discussion

It is possible that the current case report might be pathologically remarkable for two reasons at least: first, neither nodular formation nor fibrous encapsulation was evident in the tissue of spleen appearing as splenomegaly. According to the several case reports of primary HS of the spleen as summarized in Table 1, all tumors have macroscopically exhibited nodular or multi-nodular lesions with or without fibrous encapsulation, composed of a diffuse proliferation of HS cells predominantly in a sinusoidal pattern within the red pulp [1], very similar to our case. However, this case showed that the existing splenic tissue was significantly small and compressed, inducing these characteristic imaging and gross features (Figures 1 and 2), possibly compared to the other splenic HS cases [10-14]. Although we cannot provide the direct evidence that the present splenic HS shows only splenomegaly, future studies will be further required to determine how its mechanism has a role in the tumor growth after collecting and examining a larger number of splenic HS cases. Second, multiple foci of peripheral anemic infarction coexisted. It is well known that vascular invasion is a common finding in high grade malignancies, frequently associated with thrombosis and ischemia, i.e., ischemic necrosis (infarction). In spite of that, merely one splenic HS case has demonstrated relatively large necrosis due to an infarction of the splenic artery [11], as likely shown in Figure 3. As described here (Table 1), splenic HS has an extensively aggressive behavior with vessel permeation and a poor prognosis, regardless of the localized site of tumor origin. Actually, another autopsy case of HS (but entitled ‘malignant histiocytosis’ in the published paper) with unknown primary site demonstrated that the mechanical pressure elicited by severe infiltration of HS cells in the spleen should lead to capsular weakening and splenic rupture [15].

Some confusion still exists even in the recent literature with regard to the terminology of this entity, i.e., histiocytic lymphoma, malignant histiocytosis, or HS. Despite that, the term HS includes whole spectrum of localized and disseminated forms from true histiocytic lymphoma to malignant histiocytosis by definition [1-3]. In this context, it is very challenging that we pathologists strictly make a final disgnosis as primary splenic HS, since many HS cases have exhibited extensive involvement of other organs at first decision, representing as dissemination of tumor cells, such as the above splenic rupture case [15]. Indeed, so-called ‘malignant histiocytosis’ has considered to be apparently characterized by general symptoms including high fever, wasting, lymphadenopathy, hepatosplenomegaly, and progressive pancytopenia, resulting in a rapidly fatal clinical outcome, rather than localized form of HS [11,15]. Moreover, it is likely that well-defined nodular lesions are very rare in most of the ‘malignant histiocytosis’ cases [11], as in the present splenic HS. In this context, the present case seems to be categorized into classical ‘malignant histiocytosis’, rather than so-called true ‘histiocytic lymphoma’. Nevertheless, splenic HS must be a unique clinical entity, particularly associated with hypo-albuminemia, hypo-γ-globulinemia, and thrombocytopenia [11-13]. Ezdinli EZ et al. [16] proposed that, as to those mechanisms, the neoplastic histiocytes could phagocytose albumin and immunoglobulin as well as blood cells, pathologically manifesting as hemophagocytic syndrome, as shown in Figure 3B. On the other hand, HS is very likely an uncertain neoplasm from the aspects of molecular pathogenesis. Although no cytogenetic studies have been performed here, some recent papers demonstrated a clonal immunoglobulin heavy chain gene rearrangement [17,18], a clonal cytogenetic abnormality including t(14;18) [17], and a 57–80 hyperdiploid [7]/46, XY [13] karyotype, including 3 to 4 copies of various chromosomes [8]. Further studies are needed. It also remains to be elucidated whether splenectomy with or without following aggressive chemotherapy is beneficial for patients with splenic HS or not, since it has been reported that so-called ‘malignant histiocytosis’ has no or little response to splenectomy [19]. In fact, based on the current Table 1, HS of the spleen must be potentially lethal condition with short survival even after the splenectomy combined with chemotherapy and irradiation.

Pathological differential diagnoses of this splenic HS case include interdigitating dendritic cell sarcoma, follicular dendritic cell sarcoma, Langerhans cell sarcoma, diffuse large B-cell lymphoma, peripheral T-cell lymphoma, anaplastic large cell lymphoma, metastatic carcinoma, and malignant melanoma. Morphologically, tumor cells in interdigitating dendritic cell sarcoma, follicular dendritic cell sarcoma, and Langerhans cell sarcoma often have grooved, indented, folded, lobulated, or oval- to spindle-shaped nuclei, arranged in a nodular, fascicular, or storiform growth pattern, that should be absent in HS [1-3,8,9]. The conclusive diagnosis of HS is based on not only histological but immunohistochemical examination of histiocytic differentiation and exclusion of other immunophenotypes including lymphoid, epithelial, or melanocytic differentiation, as also described here (Table 2). The tumor cells in the present case apparently revealed positive expression of specific histiocytic markers, such as CD68 (KP-1), and lysozyme (Figure 4). But these HS cells also diffusely expressed the hemoglobin scavenger marker, CD163, regarded as a peculiar marker for HS as well [20].

## Conclusion

We herein reported a unique surgical case of primary splenic HS strongly associated with pathological findings of aggressive infiltration of tumor cells in the red pulp and splenic artery, in the background of splenomegaly with multiple foci of anemic infarction, but without any nodular lesions. Although splenic HS is very uncommon, not only clinicians but we pathologists should be aware that its patients undergo a fatal condition with poor prognosis, and must consider it as a differential diagnosis in cases with prolonged thrombocytopenia with splenomegaly that is non-responsive to therapy.

## Consent

Written informed consent was obtained from the patient for publication of this case report and any accompanying images. A copy of the written consent is available for review by the Editor-in-Chief of this journal.

## Competing interests

The authors declare that they have no competing interests.

## Authors’ contributions

SY and TT participated in conception of the idea and writing of the manuscript. SY, TT, NS, NA, SK, HN, KY, MT and YS performed the histological interpretation of the tumor tissue. All authors have read and approved the final manuscript.
